# Multidimensionality of the relationship between social status and dietary patterns in early childhood: longitudinal results from the French EDEN mother-child cohort

**DOI:** 10.1186/s12966-015-0285-2

**Published:** 2015-09-24

**Authors:** Soumaïla Camara, Blandine de Lauzon-Guillain, Barbara Heude, Marie-Aline Charles, Jérémie Botton, Sabine Plancoulaine, Anne Forhan, Marie-Josèphe Saurel-Cubizolles, Patricia Dargent-Molina, Sandrine Lioret, on behalf the EDEN mother-child cohort study group

**Affiliations:** INSERM, UMR1153 Epidemiology and Biostatistics Sorbonne Paris Cité Center (CRESS), Early ORigin of the Child’s Health and Development Team (ORCHAD), Paris Descartes University, France, Paris, F-75014 France; University Paris-Sud, Faculty of Pharmacy, Châtenay-Malabry, France; INSERM, UMR1153 Epidemiology and Biostatistics Sorbonne Paris Cité Center (CRESS), Obstetrical, Perinatal and Pediatric Epidemiology Team (EPOPé), Paris Descartes University, France, Paris, F-75014 France

**Keywords:** Dietary patterns, Toddlers, Preschool children, Socio-economic position, Social inequalities

## Abstract

**Background:**

The association between socioeconomic position and diet in early childhood has mainly been addressed based on maternal education and household income. We aimed to assess the influence of a variety of social factors from different socio-ecological levels (parents, household and child-care) on multi-time point dietary patterns identified from 2 to 5 y.

**Method:**

This study included 974 children from the French EDEN mother-child cohort. Two multi-time point dietary patterns were derived in a previous study: they correspond to consistent exposures to either core- or non-core foods across 2, 3 and 5 y and were labelled “Guidelines” and “Processed, fast-foods”. The associations of various social factors collected during pregnancy (age, education level) or at 2-y follow-up (mother’s single status, occupation, work commitments, household financial disadvantage, presence of older siblings and child-care arrangements) with each of the two dietary patterns, were assessed by multivariable linear regression analysis.

**Results:**

The adherence to a diet close to “Guidelines” was positively and independently associated with both maternal and paternal education levels. The adherence to a diet consistently composed of processed and fast-foods was essentially linked with maternal variables (younger age and lower education level), household financial disadvantage, the presence of older sibling (s) and being cared for at home by someone other than the mother.

**Conclusions:**

Multiple social factors operating at different levels (parents, household, and child-care) were found to be associated with the diet of young children. Different independent predictors were found for each of the two longitudinal dietary patterns, suggesting distinct pathways of influence. Our findings further suggest that interventions promoting healthier dietary choices for young children should involve both parents and take into account not only household financial disadvantage but also maternal age, family size and options for child-care.

## Introduction

A large body of epidemiological evidence shows that diets in developed countries are socially differentiated. Whether the diet is addressed food group by food group (e.g. fruits and vegetables, snack and fast foods, sweetened beverages) or as a whole through a dietary pattern approach, a positive gradient has been found between the quality of dietary intakes and socio- economic position (SEP). This has been documented at different stages during the life course, i.e. in infancy and toddlerhood [1–5], childhood and adolescence [6–8] and adulthood [7, 9]. Besides, dietary patterns have been suggested to track through infancy [2, 10, 11] into later childhood [11, 12], and from childhood to adulthood [13]. The social patterning of diet from infancy is of major public health concern: indeed, this contributes to the social patterning of child growth and development, in turn translating into social inequalities in health across the life course [14–16].

SEP in nutritional epidemiological studies involving children aged ≤5 y has mainly been addressed using maternal education, household income and, to a lesser extent, maternal occupation or employment status [1]. However, SEP encompasses other dimensions, which cannot be assumed to be interchangeable, that are likely to influence the child’s diet by distinct pathways [16, 17]. It is also possible that different dimensions of SEP influence distinct patterns of the child’s diet. The multidimensionality of the relationship between SEP and diet in children of preschool age or younger has been little studied so far [8], in contrast to adults [7, 18] and school children [6, 19–21]. In particular, paternal SEP indicators have been understudied [8]: given the increasing participation of women (including mothers) in payed occupations and the subsequent increase of fathers’ involvement in child-care and home duties, the role of paternal SEP indicators in the setting of the child’s dietary patterns is a relevant issue to be addressed.

The transition in diet across early childhood also marks important changes in terms of the social environment and educational experiences [22, 23], as the child progressively moves from the close family (in particular the parents) to the external world (care-givers, child-care, preschool). Within this changing environment, additional social factors from various socio-ecological levels [24] are also likely to impact on the child’s dietary exposures and choices. While a younger maternal age and the presence of siblings at home have been negatively related to diet quality in children ≤5 y [1, 2, 25, 26], less is known about the influence of the mother’s single status or the child-care arrangements.

A better understanding of the complexity of the relationship between diet in children aged ≤5 y and comprehensive dimensions from parental, household and child-care social environments will help to inform on the most vulnerable groups to be targeted by early prevention interventions, as well as the processes by which such interventions are more likely to be effective. We therefore aimed to assess the associations of a variety of dimensions of SEP (education, occupation, employment, household affluence), including paternal factors, and socio-demographic factors (parents’ age, maternal single status, older siblings presence and child-care arrangements), with two multi-time point dietary patterns previously identified from children 2 to 5 y in the French EDEN (Etude des Déterminants pré et post natals du développement et de la santé de l’ENfant) mother-child cohort [11].

## Methods

### Study design and participants

The EDEN mother-child cohort study aims to assess pre- and post-natal determinants of child growth, development and health [27]. In brief, between 2003 and 2006, 2002 pregnant women (<24-weeks gestation) aged 18–45 y were recruited at Nancy and Poitiers university hospitals. Exclusion criteria were twin pregnancy, history of diabetes, French illiteracy, and planning to move outside the delimited recruitment sites in the next three years. Approval for the study was obtained from the Bicêtre Hospital ethics committee (Comité Consultatif de Protection des Personnes dans la Recherche Biomédicale: ID 02-20) and the National Committee for Processed Data and Freedom (Commission Nationale de l’Informatique et des Libertés: ID 902267). Written consent was obtained from each participant.

### Measurements

Data used in the current study were collected using self-reported questionnaires completed by mothers at different stages of the follow-up. Parental and household social data were collected during pregnancy (24–28 weeks gestation) and at 2 years of follow-up, while dietary data of interest for the current study were collected when the child was aged 2, 3 and 5–6 y. Clinical records provided data on maternal age at delivery, gestational age (weeks of amenorrhea), birth weight and parity.

#### Dietary data

Children dietary intakes at 2, 3 and 5–6 y were collected using food frequency questionnaires (FFQs), described in details elsewhere [11]. These are short versions of the FFQ utilized in mothers during their pregnancy, which was validated in adults and adolescents [28]. In brief, in these short versions, the food classification was established based on similarities in food type and context of consumption and was set to be able to describe the patterns of the child diet at each age. Overall, these three FFQs included 27 food groups along with seven possible responses, ranging from “Never” to “Several times per day”, that were converted into weekly frequencies (“Never” was coded as zero, “<1 per month” as 1/8 per week, “1 to 3 times per month” as 2/4 per week, “1 to 3 times per week” as twice per week, “4 to 6 times per week” as 5 times per week, “Once a day” as 7 times per week, and “Several times per day” as 14 times per week).

The two multi-time point dietary patterns mentioned previously were identified in children by performing principal component analysis (PCA) on dietary data collected using the FFQs at 2, 3 and 5–6 y [11]. Higher scores on the “Processed, fast-foods at 2, 3 and 5 y” pattern indicated a consistent exposure to these types of foods across toddler and preschool ages, while higher scores on the “Guidelines at 2, 3 and 5 y” pattern could be interpreted as a stable adherence to a diet of high nutritional quality. These two multi-time point patterns are relevant as longitudinal measures with a high tracking on either diet.

#### Parental level social factors

##### Parental age at child’s birth

Both paternal and maternal ages at child’s birth were defined, according to the following categories: ≤24 y, 25 to 29 y, 30 to 34 y and ≥35 y.

##### Maternal single status when the child was aged 2 y

This was defined as the mother living single (yes/no).

##### Parental education

Paternal and maternal education attainments, obtained from the questionnaire completed by the mother during her pregnancy, referred to the highest diploma obtained: less than high-school, high school diploma, 2-year university degree and ≥3 year university degree.

##### Parental occupation and employment

The current job of the father when the child was aged 2 y was defined in five categories using the classification: 1) not in the labour force (student, unemployed or other inactive); 2) manual worker, shop or service worker; 3) clerical or administrative employee, farmer, craftsman or shop keeper; 4) intermediate occupation; and 5) managerial, professional or intellectual occupation. Given that mothers were more likely than fathers to be in parental leave 2 years after birth, maternal occupation status referred to the last professional activity undertaken, categorized as for fathers. Additionally, we defined maternal effective working time when the child was aged 2 y as follows: does not work, works part time and works full time; and maternal unemployment/student current status (yes/no).

#### Household level social factors

##### Household affluence when the child was aged 2 y

Household income was reported by the mother according to six intervals from: “less than 1500 euros monthly”, to “more than 4500 euros monthly”. Perceived financial hardship over the last year to purchase each of the following goods for the family: food, clothes, accommodation related bills, or medical care/drugs, were dichotomised in yes/no. Bank overdraft frequency over the last year was also dichotomised into: never or occasionally *vs.* several times or often. Two additional variables related to health cover: being eligible (yes/no) for French social welfare health insurance (Couverture Maladie Universelle), an indicator of disadvantage, and subscription to a private supplementary health insurance (yes/no).

##### Presence of any older sibling at home

This variable was dichotomised in yes/no.

#### Child-care level social factors

##### Child-care arrangements

This variable was defined in four categories: crèche or preschool (the latter starting in France at 2–3 y), at a nanny’s home, cared for at home by the mother, and cared for at home by someone other than the mother.

#### Missing data

Missing SEP data at 2 y were substituted in <5 % of parents by the value of the same variable reported at other times of follow-up either at 1 y, 3 y (if not available at 1 y follow-up), or during pregnancy (if not available at 3 y follow-up).

#### Population studied

Of the 2002 women initially recruited, 1899 children were eligible for the EDEN cohort at birth. Multi-time point dietary patterns were previously assessed in the 989 children with complete dietary data at ages 2, 3 and 5–6 y [11]. Fifteen children were excluded due to missing values in the social factors studied, resulting in a sample of 974 children for the current study.

#### Statistical analyses

We describe the children from the EDEN cohort who had all social and dietary data available (*n* = 974). Socio-demographic factors were then compared between this sample and the children eligible at birth but not included in the analysis because of dropping out or missing data (*n* = 925). Chi-square and Student - *t* tests were used to compare frequencies and means, respectively.

PCA was used to synthesise the eight household standardized variables, namely: income, perceived hardship over the past year to purchase four different types of goods for the family, bank overdraft frequency over the past year, and the two variables relating to health cover. The number of patterns was selected considering eigenvalues >1.0, the scree plot and the interpretability of the patterns [29, 30]. To interpret the results and provide a label to a given pattern, we considered the items most strongly related to that pattern, i.e. those for which the absolute value of the loading coefficient (which is the correlation of each variable with the given pattern) was >0.30. The pattern scores were calculated at the individual level by summing the observed standardized household variables, weighted according to the PCA loadings.

The associations between the multi-time point dietary patterns (two continuous dependent variables) and social factors were analysed by multivariable linear regression, first adjusted for a basic set of variables, namely the child’s age and gender, recruitment centre and the season when the food frequency questionnaire at age 2 was completed (referred to as Model 1); then mutually adjusted for all social factors (referred to as Model 2). We checked the absence of a significant moderation effect by gender, as well as the absence of multicollinearity, assessed with the variance inflation factor criterion (VIF < 5) [31].

The significance level was set at 5 %. Values in the text are either percentages (%) or means ± SD. Analyses used SAS software (version 9.3).

## Results

### Characteristics of the study population

Our population characteristics are presented in Table 1. While children excluded from the analysis did not differ from the children included, according to sex, prematurity, and birth weight, they were more likely to be born to younger mothers (28.8 y ± 5.1 *vs.* 30.2 y ± 4.6, *P* = 0.0009) and fathers (31.5 y ± 6.4 *vs.* 32.3 y ± 5.5, *P* = 0.007), mothers who were single when pregnant (4.3 % *vs*. 0.3 %, *P* < 0.0001), and multiparous mothers (59.4 % vs. 51.9 %, *P* = 0.0009). Also, mothers and fathers were less likely to have a University degree (42.3 *vs*. 62.1 %, and 35.7 vs. 51.0 %, respectively, *P* < 0.0001).

**Table 1:** Characteristics of the study population (values are % yes or means ± SDs unless otherwise indicated, *n* = 974). The EDEN mother-child cohort

	Value
Child characteristics	
Male gender	53.4
Premature birth (<37 weeks of amenorrhea)	5.8
Birth weight, g^a^	3295 ± 505
Paternal characteristics	
Age at delivery, y	
≤24	5.5
25–29	24.0
30–34	41.2
≥35	29.3
Education	
< high-school	28.0
High school diploma	20.9
2-year university degree	22.4
≥3-year university degree	28.6
Current occupation^b^	
Not in the labour force	4.6
Manual worker, shop or service worker	28.3
Employee, farmer, craftsman or shop keeper	18.6
Intermediate occupation	29.6
Managerial, professional or intellectual occupation	18.9
Maternal characteristics	
Age at delivery, y	
≤24	9.8
25–29	35.6
30–34	37.3
≥35	17.4
Education	
< high-school	20.5
High school diploma	17.4
2-year university degree	24.1
≥3-year university degree	38.0
Current or past occupation^b^	
Not in the labour force	3.5
Manual worker, shop or service worker	16.3
Clerical or administrative employee, farmer, craftswoman or shop keeper	25.3
Intermediate occupation	41.9
Managerial, professional or intellectual occupation	13.0
Working time^b^	
Does not work	28.4
Works part time	30.0
Works full time	41.6
Unemployed or student^b^	5.7
Single status of the mother^b^	2.3
Household characteristics	
Monthly household income^b^, €	
≤1500	7.6
1501–2300	23.9
2301–3000	29.5
3001–3800	21.4
3801–4500	9.8
≥4501	7.9
Hardship over the past year to purchase foods for the family^b,c^	6.6
Hardship over the past year to purchase clothes for the family^b,c^	8.0
Hardship over the past year to pay accommodation related bills^b,c^	9.7
Hardship over the past year to access medicalcare/drugs^b,c^	1.3
Bank overdraft frequency over the past year^b^	
Never or occasionally	75.1
Several times or often	24.9
Being eligible for French social welfare health insurance^b,d^	3.9
Subscription to a private supplementary health insurance^b^	97.1
Child social environment	
Older siblings at home^b^	21.1
Child-care arrangements^b^	
Crèche or pre-school	21.8
At nanny’s home	45.9
At home, but not cared for by mother	10.1
At home, cared for by mother	22.3

Values are % yes or mean ± SD, *n* = 974

^a^Average birth weight was 3231 ± 584 g in the French national perinatal survey performed in 2003 (http://www.drees.sante.gouv.fr/IMG/pdf/enp_2003_rapport_inserm.pdf)

^b^When child is aged 2 y

^c^Yes, some or important hardship

^d^“Couverture Maladie Universelle”

### Household affluence

The first pattern identified from PCA accounted for 34.3 % of the explained variance (Table 2) and was retained as a measure of household affluence. It was positively correlated with perceived financial hardships to purchase food and clothes, pay bills and access medical care/drugs, as well as with bank overdraft frequency and eligibility for French social welfare health insurance; and had negative loadings for household income and subscription to a private supplementary health insurance. We labelled this pattern “Household disadvantage composite index”.

**Table 2:** PCA loadings for the household affluence pattern (1^st^ pattern) derived when child was aged 2 y. The EDEN mother-child cohort

Household indicators of affluence	PCA loadings
Monthly income	−0.54
Hardship to purchase foods for the family	0.76
Hardship to purchase clothes for the family	0.71
Hardship to pay accommodation related bills	0.74
Hardship to access medical care/drugs	0.46
Bank overdraft frequency over the past year	0.53
Being eligible for French social welfare health insurance	0.41
Subscription to a supplementary health insurance	−0.39
% of variance explained	34.3
Label	“Household disadvantage” pattern

### Associations of social factors with multi-time dietary patterns

#### “Processed, fast-foods at 2, 3 and 5 y” dietary pattern

With the exception of two variables (namely maternal unemployment/student status and single status), all social factors under study were significantly associated with the previously identified [11] “Processed, fast-foods at 2, 3 and 5 y” dietary pattern in the model with basic adjustments (Model 1) (Table 3). The only factors that remained significantly associated with this dietary pattern in the fully-adjusted model (Model 2) were maternal age and education, as well as the household disadvantage composite index, the presence of older siblings at home and child-care arrangements.

**Table 3:** Coefficients (SE) of the multivariable linear regression analyses, with the multi-time point dietary patterns as the dependent variables. The EDEN mother-child cohort

	“Processed, fast-foods at 2, 3 and 5 y” dietary pattern scores		“Guidelines at 2, 3 and 5 y” dietary pattern scores	
	Model 1^a^	Model 2^b^	Model 1^a^	Model 2^b^
Paternal factors				
Age at delivery, y				
<=24	0.49 (0.15)***	−0.14 (0.18)	−0.21 (0.15)	0.07 (0.18)
25–29	−0.06 (0.09)	−0.20 (0.10)	−0.13 (0.09)	−0.14 (0.11)
30–34	0.00 (0.08)	−0.08 (0.08)	−0.11 (0.08)	−0.14 (0.08)
> = 35	0	0	0	0
*P*-value	0.003	0.30	0.30	0.22
Education level				
< high-school	0.45 (0.08)***	0.03 (0.12)	−0.66 (0.08)***	−0.38 (0.12)**
High school diploma	0.32 (0.09)***	0.05 (0.11)	−0.43 (0.09)***	−0.19 (0.12)
2-year university degree	0.08 (0.09)	−0.02 (0.10)	−0.35 (0.09)***	−0.25 (0.10)*
≥3-year university degree	0	0	0	0
*P*-value	< 0.0001	0.92	< 0.0001	0.008
Current occupation when child aged 2 y				
Not in the labour force	0.26 (0.16)	−0.22 (0.18)	−0.87 (0.16)***	−0.44 (0.18)*
Manual worker, shop or service worker	0.50 (0.09)***	0.12 (0.12)	−0.57 (0.09)***	−0.10 (0.13)
Employee, farmer, craftsman or shop keeper	0.23 (0.10)*	0.04 (0.12)	−0.42 (0.10)***	−0.09 (0.12)
Intermediate occupation	0.10 (0.09)	0.00 (0.10)	−0.27 (0.09)***	−0.06 (0.11)
Managerial, professional or intellectual occupation	0	0	0	0
*P*-value	< 0.0001	0.27	< 0.0001	0.19
Maternal factors				
Age at delivery, y				
<=24	0.57 (0.13)***	0.57 (0.16)***	−0.19 (0.13)	0.01 (0.16)
25–29	0.07 (0.09)	0.30 (0.11)**	−0.03 (0.09)	0.03 (0.11)
30–34	0.09 (0.09)	0.21 (0.10)*	0.08 (0.09)	0.11 (0.10)
> = 35	0	0	0	0
*P*-value	*p* < 0.0001	0.004	0.11	0.56
Education level				
< high-school	0.75 (0.09)***	0.50 (0.11)***	−0.63 (0.09)***	−0.33 (0.12)**
High school diploma	0.29 (0.09)**	0.11 (0.11)	−0.39 (0.09)***	−0.14 (0.11)
2-year university degree	0.13 (0.08)	0.10 (0.09)	−0.18 (0.08)*	−0.03 (0.09)
≥3-year university degree	0	0	0	0
*P*-value	< 0.0001	< 0.0001	< 0.0001	0.027
Current or past occupation when child aged 2 y				
Not in the labour force	0.48 (0.19)*	−0.11 (0.21)	−0.29 (0.19)	0.18 (0.21)
Manual worker, shop or service worker	0.62 (0.12)***	−0.03 (0.14)	−0.53 (0.12)***	0.00 (0.15)
Employee, farmer, craftswoman or shop keeper	0.26 (0.11)*	−0.10 (0.12)	−0.41 (0.11)**	−0.02 (0.13)
Intermediate occupation	0.11 (0.10)	0.00 (0.10)	−0.10 (0.10)	0.05 (0.11)
Managerial, professional or intellectual occupation	0	0	0	0
*P*-value	< 0.0001	0.82	< 0.0001	0.79
Working time when child aged 2 y				
Does not work	0.27 (0.08)***	0.20 (0.12)	−0.13 (0.08)	0.05 (0.12)
Works part time	0.05 (0.08)	0.00 (0.07)	0.02 (0.08)	0.03 (0.08)
Works full time	0	0	0	0
*P*-value	0.002	0.24	0.15	0.89
Unemployed or student when child aged 2 y				
No	0	0	0	0
Yes	0.17 (0.14)	0.01 (0.14)	−0.15 (0.14)	−0.10 (0.15)
*P*-value	0.22	0.96	0.29	0.50
Single				
No	0	0	0	0
Yes	0.21 (0.21)	−0.05 (0.21)	−0.41 (0.21)	−0.22 (0.22)
*P*-value	0.33	0.82	0.06	0.30
Household factors when child aged 2 y				
Household disadvantage composite index	0.20 (0.04)	0.10 (0.04)	−0.17 (0.04)	−0.06 (0.04)
*P*-value	< 0.0001	0.008	< 0.0001	0.14
Social environment when child aged 2 y				
Older sibling at home				
No	0	0	0	0
Yes	0.26 (0.08)	0.20 (0.08)	−0.07 (0.08)	−0.02 (0.08)
*P*-value	0.0008	0.018	0.39	0.80
child-care arrangements				
Crèche or preschool	−0.36 (0.09)***	−0.03 (0.13)	0.21 (0.10)*	0.08 (0.13)
At a nanny’s home	−0.20 (0.08)*	0.13 (0.13)	0.16 (0.08)	0.10 (0.13)
At home, but not cared for by mother	0.54 (0.12)***	0.70 (0.14)***	−0.10 (0.12)	0.01 (0.15)
At home, cared for by mother	0	0	0	0
*P*-value	< 0.0001	< 0.0001	0.02	0.82

*p < 0.05; **p < 0.01; ***p < 0.001

^a^Model 1 includes only one of the social factors listed in this table and adjusts for the child’s age and gender, recruitment center and season

^b^Model 2 includes all social factors listed in this table and adjusts for the child’s age and gender, recruitment center and season

#### “Guidelines at 2, 3 and 5 y” dietary pattern

With the exception of maternal unemployment/student status and maternal working time, all maternal and paternal SEP variables were positively associated with adherence to the “Guidelines at 2, 3 and 5 y” pattern in the basic model (Model 1) (Table 3). Higher scores were also observed in children enrolled in day care provided by crèches or preschools. The only two predictors remaining independently associated in the fully-adjusted model (Model 2) were paternal and maternal education levels.

## Discussion

To our knowledge this is the first study to prospectively explore the multidimensionality of the association between social status (including paternal, maternal, household and child-care factors) and dietary patterns in toddler and preschool ages. Two complementary analytical approaches were undertaken. The first addressed each social factor separately, allowing us to examine the issue of social inequalities in early diet across a variety of dimensions. The second accounted for all social factors simultaneously, shedding light on the social determinants that are independently associated with tracking across early childhood of non-core foods and of foods closer to nutritional guidelines.

### Social inequalities in diet across early childhood

The EDEN mother-child cohort is population-based, which allowed us to investigate the extent to which diet across toddler and preschool ages was associated with a social gradient. Although the most disadvantaged households were under-represented in the current study, as is often the case in longitudinal studies, the analysis of the social factors associated with each of the two multi-time point dietary patterns (Model 1) confirmed the positive social gradient of diet with almost all SEP indicators under study, i.e. education and occupation (both paternal and maternal), and the pattern of household financial disadvantage. The size and consistency of this gradient across a variety of dimensions add evidence towards the proposition that diet in early childhood is already strongly socially patterned.

These findings therefore inform on the social characteristics that can be screened in the context of early childhood nutrition interventions, to identify subgroups at higher risk of lower quality diets. While young mothers with low educational attainments and/or low income have already been identified as a relevant target in a number of studies [1–6, 20], further attention could also be warranted towards young fathers with a lower education and/or lower occupational status, as well as families with young children displaying any form of insecurity (e.g. with regards to the provision of essential needs).

#### Independent associations between social determinants and multi-time point dietary patterns

Our findings further suggest that distinct social determinants influenced different patterns of diet. The adherence to a diet close to “Guidelines” across toddler and preschool ages was positively and independently associated with both maternal and paternal education. The adherence to a diet consistently composed of processed and fast-foods seemed to be essentially determined by maternal variables (i.e. younger age and lower education level), household financial disadvantage, the presence of older sibling (s) and less structured child-care arrangements.

Consistent with studies involving school-aged children [6, 20] and children aged ≤5 y [1–5, 32], our results confirmed the major positive association of maternal education with healthier dietary patterns across early childhood. The mediating factors suggested for the relationship between maternal education and the child’s diet in the general paediatric literature include maternal nutrition knowledge; positive concern and attitudes of the mother towards healthy eating, self-efficacy, feeding practices and modelling, as well as the quality of foods made available and accessible at home [20]. Of note, in the Melbourne Infant Feeding Activity and Nutrition Trial (InFANT) Program, maternal nutrition knowledge mediated the association between maternal education and the mothers’ diet [33], while the latter was suggested to mediate the association between maternal education and infant dietary patterns [4]. Maternal modelling of infants’ and toddlers’ diet has also been suggested in a number of other reports [10, 32, 34].

Far scarcer are studies addressing fathers’ social influence on their child’s diet [8, 19], and even rarer are studies focused on children ≤5 y [1]. Based on the European IDEFICS Study, Fernandez-Alvira et al. [8] also showed that maternal and paternal education attainments were independently and positively associated with the persistence of membership to the ‘Healthy’ dietary pattern (derived using cluster analysis) between baseline (children aged 2–9 y) and follow-up (2 years later). Likewise, a positive relationship between the ‘Healthy’ dietary pattern, derived from PCA in a sample of 2 year-old Norwegian toddlers, was also found with paternal education using multivariable analysis [26]. This evidence supports the hypothesis that maternal and paternal educations are not proxies for one another, but rather independent determinants. It is likely that mediators previously attributed to mothers are shared by fathers, e.g. modelling [35]. Interestingly, the paternal educational influence only remained in the multivariable model explaining the “Guideline” dietary pattern: independently of financial resources, the adherence to a healthy diet over toddlerhood and preschool ages thus appears as more demanding in terms of parental support and is enhanced when both parents exert their educational influence. This particular finding also suggests that even at a higher level of disadvantage, providing both mothers and fathers with more knowledge and concern towards healthy eating is likely to be a means of improving their feeding practices, and subsequently their child’s diet.

Whether the diet of children aged ≤5 y is associated with household affluence is debated, in particular when household income stands as a proxy for wealth [1, 17]. Household affluence encompasses other dimensions such as those we incorporated in our composite index: we found that the resulting “Household disadvantage composite index” assessed at 2 y was predictive of a consistent exposure to non-core foods over the three subsequent years. Similar findings concerning 3 year-old children from the Avon Longitudinal Study of Pregnancy and Childhood (ALSPAC) are worth highlighting [36]. In this study, a measure of the degree of financial difficulty was estimated based on a list of five items (food, clothing, heating, rent/mortgage, things for child) that the mother may have had trouble affording. Based on multivariable models accounting for a range of socio-demographic and SEP factors, the authors also observed higher scores on their so called “Junk” dietary pattern in children living in households presenting higher financial hardship. Energy-dense and nutrient-poor diets have been shown to have a lower cost per mega joule [7], which could drive such relationships in the context of constrained budget, independently of education background.

Though children of fathers not in the labour force displayed lower scores on the “Guidelines” pattern, occupation did not remain significantly associated with either multi-time point dietary pattern in the multivariable model (Model 2), consistent with previous observations [32]. These findings suggest that occupation (as compared to education) is less likely to influence knowledge on health, nutrition and feeding practices. Education may also better reflect parent’s capacity to access, interpret and put into practice health information [17, 37]. With respect to maternal employment, few studies [1, 36, 38], but not all [39], have reported that children of mothers working full time had rather unhealthier diets, as compared to children of non-working mothers who presumably have more time to allocate to food (such as grocery shopping, cooking, eating with the child), but these associations were not seen in our study population.

Consistent with previous studies in children ≤5 y, higher scores on the multi-time point “Processed, fast-foods” pattern were observed in younger mothers and in the presence of older siblings [1, 2, 25, 26]. Generational differences in lifestyle or lower concern with respect to healthy eating behaviours in younger mothers have been suggested [40]. Older siblings are more exposed to snacks or sweetened beverages, given their larger social network. By favouring the introduction of less healthy foods in the home, and therefore increasing exposure and facilitating access to these particular items, older siblings are likely to challenge the parent’s willingness to follow dietary guidelines [10, 32]. Modelling older brothers and sisters is also possible. Besides, we cannot exclude the possibility that parents of larger families have overall less time available to purchase and cook healthy foods.

As in other studies [32, 36], but not all [25], our analysis did not confirm the negative effect of the single status of the mother on the child’s diet, which could be partly explained by the relative small number of single mothers in the analysed sample. Beyond the familial environment, our findings further suggest that toddlers cared for at home at age 2 y by someone other than the mother had higher scores on the “Processed, fast-foods at 2, 3 and 5 y” dietary pattern, as compared to their counterparts enrolled in crèche or preschool, or those cared for by the mother. Comparison with other findings is not straightforward, given the paucity of studies on this issue. French crèches and preschools are supposed to provide eating routines and meals in accordance with nutritional guidelines; while further research is needed, we can hypothesise that this particular environment may prevent young children from being too frequently exposed to unhealthy foods. The fact that toddlers cared for at home by someone other than the mother had higher scores on the “Processed, fast-food” pattern is difficult to explain and requires further investigation.

#### Limitations and strengths

While women from all SEPs were recruited at baseline, university-educated parents were over-represented in the sample analysed. This typical feature of cohort studies may limit generalisability of our results, thus we cannot exclude the possibility that the relationships under study would differ in samples where disadvantaged families are better represented. We may however hypothesise that the social gradient would be even steeper if all social groups were better represented in the study at the 2 y follow-up. We also acknowledge that the measurements of social status addressed here do not encompass all levels of the socio-ecological model. For example parental cultural origin was not accounted for, due to too small numbers in these groups, while at a more distal level, area deprivation was not included in the analysis. Our results however confirmed a social gradient for a wide range of social factors, including paternal characteristics, providing robustness and original insights into the issue of social inequalities in diet in early childhood. While the social situation of parents (in particular that of mothers) is likely to change substantially upon the birth of a child, a further strength of our study was to account for social factors assessed when the child was aged 2 y, thus allowing the associations with dietary patterns spanning 2 to 5 y to be assessed prospectively.

## Conclusion

These findings confirm that when studying early diet from a social perspective, one size does not fit all [17]: the choice of socioeconomic or socio-demographic indicators used does matter [21]. Multiple social factors operating at different levels (parents, household, child-care) seem to influence distinct aspects of diet, highlighting the complex embedding and cumulative nature of disadvantage. These results confirmed the major association of early dietary patterns with maternal education. They also highlight that beyond household financial disadvantage, the social environment of the child (family composition and day-care) contributes to the orientation of the diet towards either core or non-core foods. The child’s adherence to a healthy diet on a regular basis also results from paternal influences, which indicates that fathers play an important role in the setting of healthy dietary habits in their children. Our findings therefore suggest that interventions promoting healthier dietary choices for children should involve both parents, target younger parents and more specifically socially disadvantaged families, and take family composition as well as options for child-care into account. To prevent cumulative effects of social adversity over the life course, such complex interventions are likely to be more effective in reducing social inequalities if started as soon as pregnancy and early childhood.

## Abbreviations

EDEN: Etude des Déterminants pré et post natals du développement et de la santé de l’ENfant)
FFQ: Food Frequency Questionnaire
PCA: Principal component analysis
